# Supplementary material for: Methylation Patterns of the FKBP5 Gene in Association with Childhood Maltreatment and Depressive Disorders
Source: Int J Mol Sci. 2024 Jan 25;25(3):1485. doi: 10.3390/ijms25031485 (PMC10855893; doi:10.3390/ijms25031485)
Supplement: Supplementary file 1 [file ijms-25-01485-s001.zip › ijms-2814057-supplementary.pdf]

Supplement:

S1) Figures

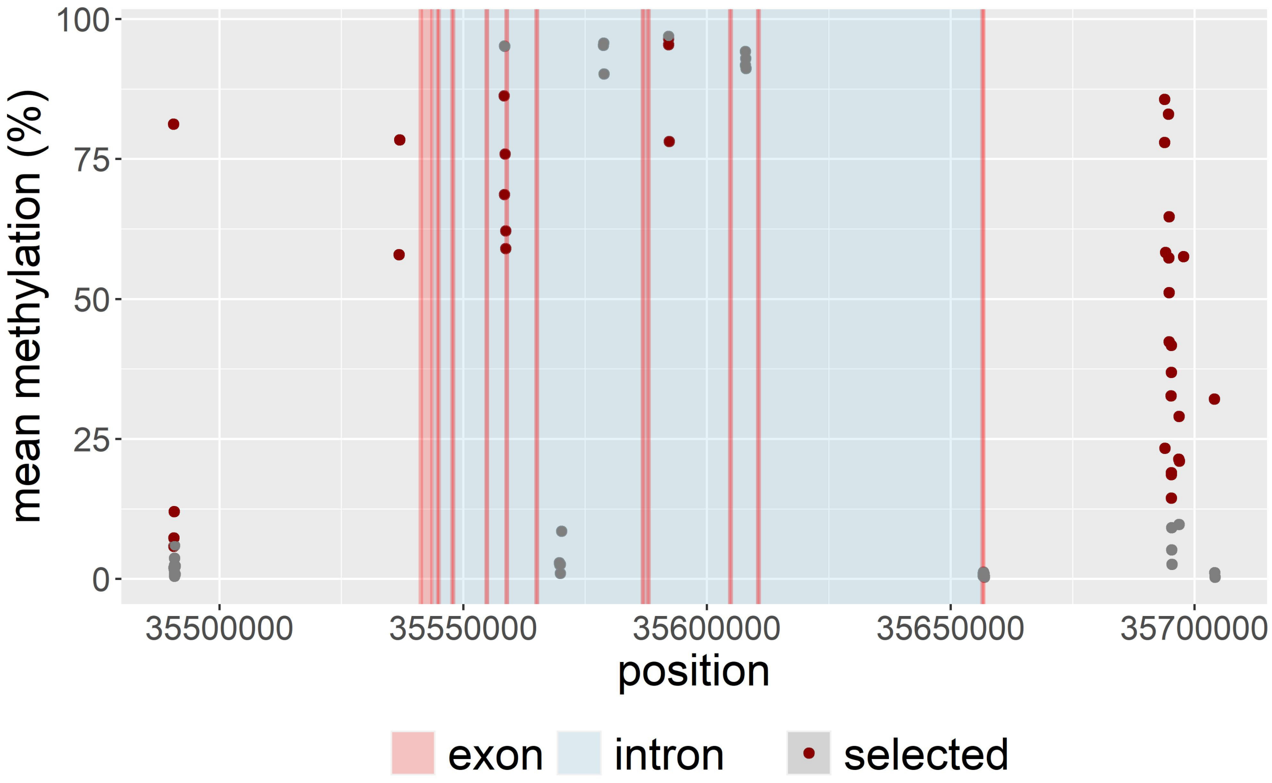

Figure S1. *Mean methylation of the FKBP5 gene (hg19. chr6:35695327-35695346) for each CpG site across all participants. Dark red points mark the 34 CpGs that passed the selection criteria.*

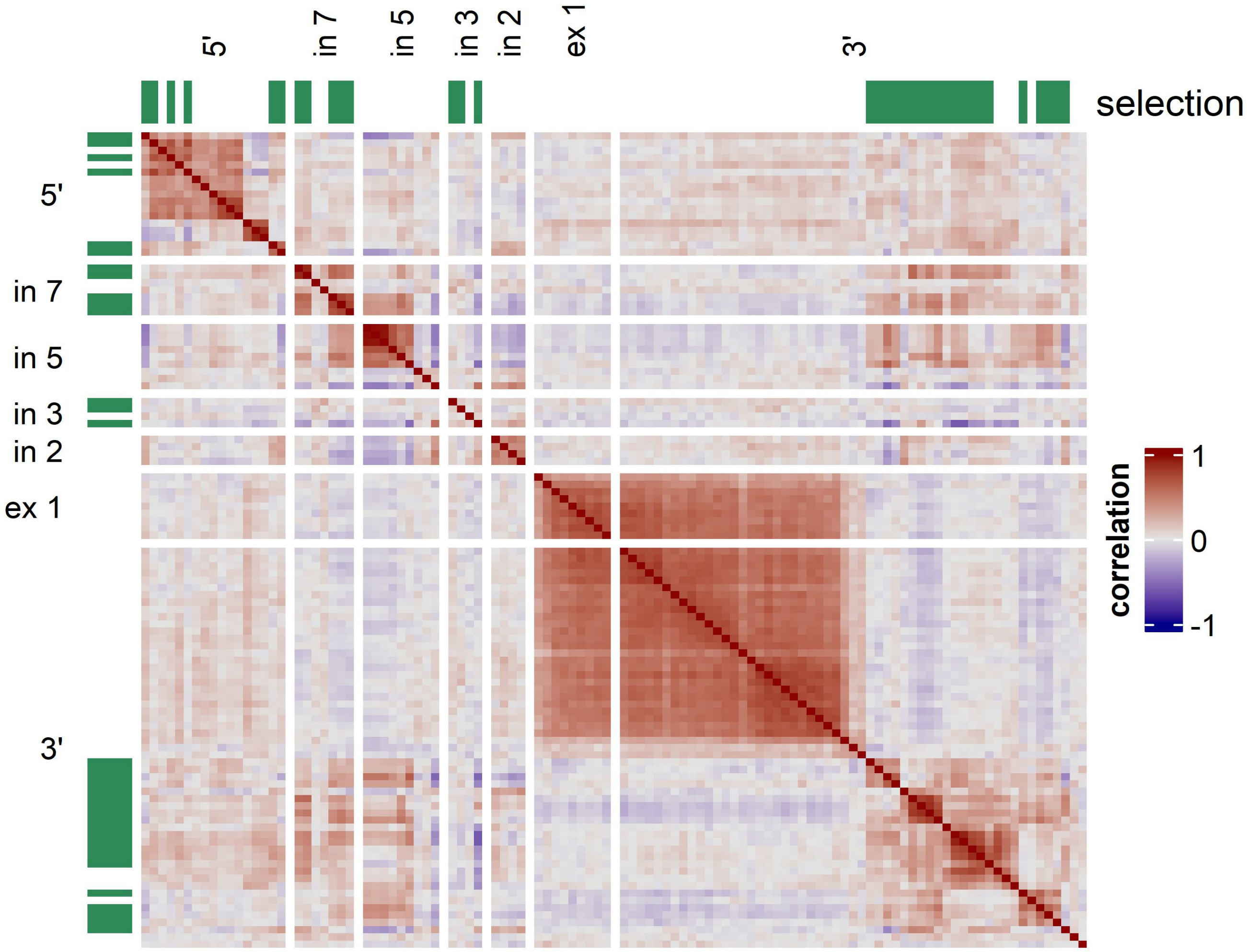

Figure S2. *Correlation coefficients of the methylation levels across the FKBP5 CpG sites for introns (int), exons (ex), and 3'-/5'-end; green bars mark the subset of 34 CpGs in the focus of our study.*

S2) Power analysis

To assess the power of our study, we estimated the observable effect size depending on the sample size for a desired power of 0.95 using G*Power[1]. Since the results were similar for 15-20 predictors, the plot for 20 predictors (as in the gene expression model) is shown as an example in Figure S3. Note that the method does not account for random effects and thus is only an estimation of true power.

For 203 participants, as in our sample, only effects above 0.1 may be detected with a power of 0.95 and a significance level of 0.05/34=0.0014 for 34 tests. To detect small effect sizes (Cohen's f < 0.1), a sample size of 500-1000 participants is required.

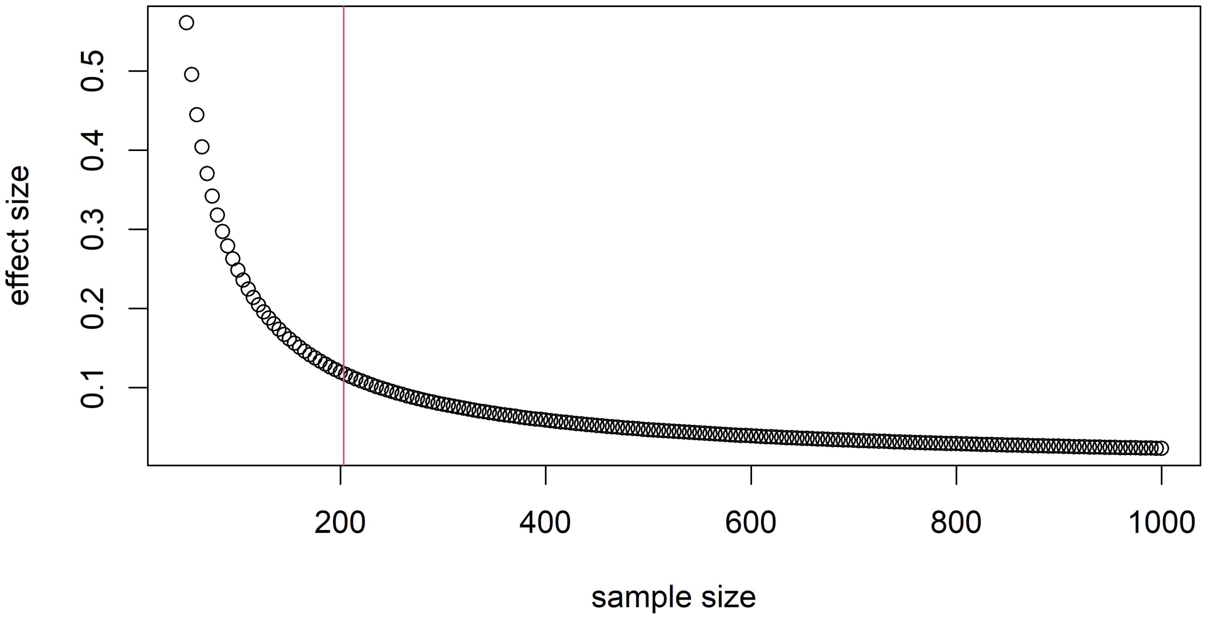

Figure S3. *Power = 0.95; significance level of 0.00147; the red line marks the given sample size of 203 participants.*

[1] Faul, F., Erdfelder, E., Lang, A.-G., & Buchner, A. (2007). G*Power 3: A flexible statistical power analysis program for the social, behavioral, and biomedical sciences. Behavior Research Methods, 39, 175-191.

S3) Quality control and CpG selection

Histograms of *M*-values for single CpGs were plotted to check whether approximation with a normal distribution is plausible. Three histograms indicate a grouping pattern of higher and lower methylated samples: CpGs 35490608 and 35490619, which are highly correlated (r=0.93), as well as CpG 35694577 (Figure S4a). Using scatterplots, associations between covariates and grouping effects of M-values were inspected, but none of the covariates explained the effect (see Figures S4b-c as examples). Hence, these findings are treated as artefacts, and the three CpGs were excluded from further analyses.

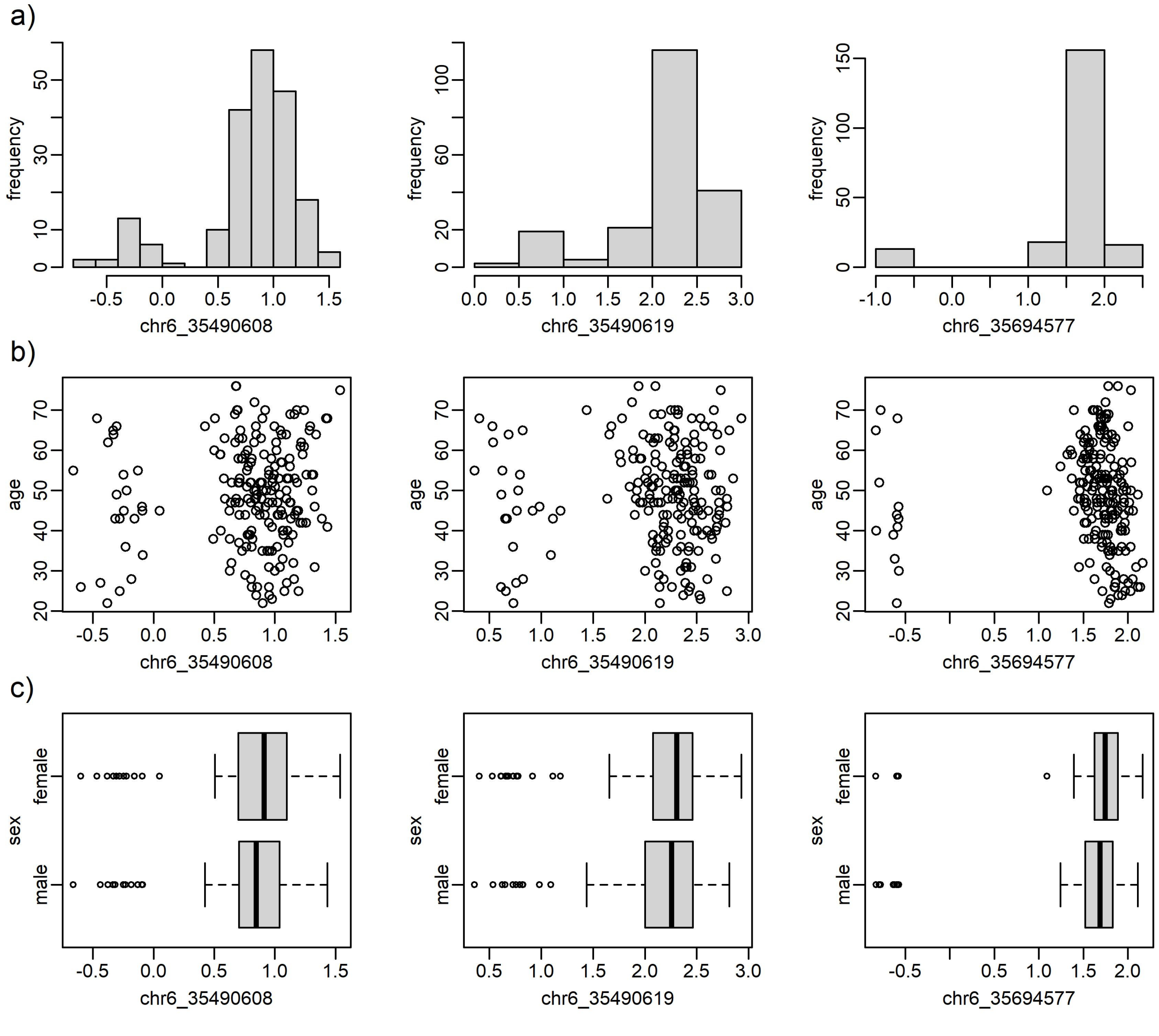

***Figure S4**. Visual inspection of three CpG sites with atypical M-value distribution; (a) histogram of methylation M-values; (b) scatterplot of methylation M-values vs. age; (c) boxplots of sex-stratified M-value distributions.*

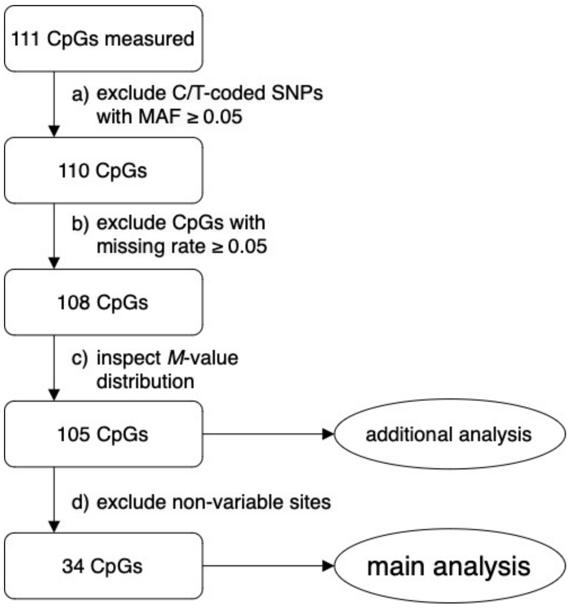

Figure S5. *Workflow of CpG site selection: a) C/T-coded SNPs with minor allele frequency (MAF) ≥ 0.05 were excluded; b) CpGs with missing rate ≥ 0.05 were excluded; c) CpGs that displayed an atypical distribution of M-values were excluded by visual inspection; d) non-variable CpGs where the methylation beta values of all individuals were <20% or >80% were excluded from the main analysis.*

S4) Genotyping

A subset of the SHIP-TREND samples was genotyped using the Illumina Human Omni 2.5 array. Nonfasting blood samples were drawn from the cubital vein in the supine position. The samples were taken between 07:00 AM and 04:00 PM, and serum aliquots were prepared for immediate analysis and for storage at -80 °C in the Integrated Research Biobank (Liconic, Liechtenstein). DNA from whole blood was prepared using the Gentra Puregene Blood Kit (Qiagen, Hilden, Germany) according to the manufacturer's protocol. The purity and concentration of DNA were determined using a NanoDrop ND-1000 UV-Vis Spectrophotometer (Thermo Scientific). The integrity of all DNA preparations was validated by electrophoresis using 0.8% agarose-1x TBE gels stained with ethidium bromide. Subsequent sample processing and array hybridization was performed as described by the manufacturer (Illumina, San Diego, CA, USA) at the Helmholtz Zentrum München (Germany). Genotypes were called with the GenCall algorithm of GenomeStudio Genotyping Module v1.0. Arrays with a genotyping call rate <94%, duplicates (based on estimated IBD), and mismatches between reported and genotyped sex were removed, leaving 986 arrays for subsequent analyses. Imputation of genotypes was performed using the HRCv1.1 reference panel and the Eagle and minimac3 software implemented in the Michigan Imputation Server for pre-phasing and imputation, respectively. SNPs with a

Hardy–Weinberg equilibrium p-value <0.0001, a call rate <0.95, or monomorphic SNPs were removed before imputation, as well as SNPs with a position mapping problem from genome build b36 to b37, duplicate IDs, or with inconsistent reference site alleles. rs1360780 was imputed with imputation quality = 99.7% and a minor allele frequency (MAF) = 31%.

S5) Tables

Main models

***Table S1**: Linear mixed model results for FKBP5 methylation on FKBP5 expression: genomic position, estimate of the regression coefficient, standard error, t-value, degrees of freedom, and p-value.*

CpG (chr6)	Est.	S.E.	*t* val.	d.f.	*p* val.
chr6:35490599	0.037	0.107	0.346	178	0.73
chr6:35490654	0.063	0.05	1.254	175	0.212
chr6:35490693	0.006	0.051	0.126	175	0.9
chr6:35490744	0.059	0.055	1.072	176	0.285
chr6:35536880	-0.014	0.113	-0.126	172	0.9
chr6:35536999	0.046	0.1	0.462	177	0.645
chr6:35558386	0.036	0.076	0.466	176	0.641
chr6:35558438	-0.04	0.078	-0.518	175	0.605
chr6:35558566	-0.039	0.103	-0.376	177	0.708
chr6:35558710	0.043	0.102	0.42	177	0.675
chr6:35558721	0.067	0.099	0.671	177	0.503
chr6:35592135	0.188	0.109	1.715	175	0.088
chr6:35592141	-0.151	0.101	-1.494	174	0.137
chr6:35592235	0.016	0.095	0.167	177	0.868
chr6:35693873	-0.077	0.067	-1.163	177	0.247
chr6:35693881	-0.054	0.057	-0.95	175	0.344
chr6:35693949	-0.133	0.065	-2.054	174	0.041
chr6:35694056	-0.088	0.078	-1.122	175	0.263
chr6:35694626	-0.039	0.131	-0.298	178	0.766
chr6:35694724	-0.106	0.103	-1.027	174	0.306
chr6:35694748	-0.086	0.104	-0.825	177	0.41
chr6:35694756	-0.084	0.1	-0.839	175	0.403
chr6:35694787	-0.12	0.123	-0.971	178	0.333
chr6:35695192	-0.052	0.074	-0.698	176	0.486
chr6:35695226	-0.097	0.11	-0.883	177	0.379
chr6:35695240	-0.018	0.103	-0.179	176	0.858
chr6:35695267	-0.032	0.096	-0.333	176	0.739
chr6:35695270	0.032	0.092	0.354	177	0.724
chr6:35695273	0.079	0.058	1.349	177	0.179
chr6:35696726	-0.033	0.073	-0.457	176	0.648
chr6:35696823	-0.078	0.082	-0.962	177	0.337
chr6:35696870	-0.094	0.082	-1.143	176	0.255
chr6:35697759	-0.07	0.052	-1.358	177	0.176
chr6:35704069	-0.01	0.127	-0.082	178	0.934

Remaining CpGs not considered in the results of this study

CpG (chr6)	Est.	S.E.	*t* val.	d.f.	*p* val.
chr6:35490674	0	0.034	-0.007	175	0.995
chr6:35490713	-0.036	0.033	-1.112	174	0.268
chr6:35490782	-0.029	0.029	-1.006	178	0.316
chr6:35490787	-0.008	0.033	-0.249	177	0.804
chr6:35490800	-0.036	0.036	-0.988	178	0.324
chr6:35490812	-0.004	0.041	-0.108	177	0.914
chr6:35490818	-0.003	0.055	-0.063	177	0.95
chr6:35490820	-0.003	0.058	-0.055	177	0.956
chr6:35490925	0.01	0.03	0.347	174	0.729
chr6:35490946	-0.032	0.028	-1.115	174	0.266
chr6:35490965	-0.001	0.027	-0.024	173	0.981
chr6:35558488	0.123	0.095	1.288	178	0.199
chr6:35558513	0.13	0.096	1.356	176	0.177
chr6:35569751	-0.054	0.046	-1.19	177	0.236
chr6:35569757	-0.044	0.042	-1.05	176	0.295
chr6:35569777	-0.055	0.045	-1.225	177	0.222
chr6:35569896	0.009	0.041	0.21	176	0.834
chr6:35569922	-0.025	0.054	-0.46	177	0.646
chr6:35570224	-0.26	0.083	-3.147	178	0.002
chr6:35578739	0.256	0.144	1.782	177	0.076
chr6:35578830	0.211	0.121	1.742	178	0.083
chr6:35578891	0.026	0.135	0.195	174	0.846
chr6:35592151	-0.053	0.111	-0.478	173	0.633
chr6:35607856	0.065	0.126	0.515	173	0.608
chr6:35607904	-0.078	0.112	-0.692	175	0.49
chr6:35607969	-0.016	0.114	-0.144	177	0.886
chr6:35608022	-0.115	0.124	-0.927	177	0.355
chr6:35656649	-0.052	0.031	-1.681	176	0.094
chr6:35656661	-0.019	0.026	-0.723	176	0.471
chr6:35656665	-0.03	0.03	-0.99	176	0.323
chr6:35656667	-0.011	0.03	-0.381	174	0.703
chr6:35656669	-0.04	0.029	-1.352	176	0.178
chr6:35656672	-0.011	0.025	-0.448	173	0.654
chr6:35656679	-0.005	0.029	-0.159	174	0.874
chr6:35656682	-0.009	0.03	-0.315	175	0.753
chr6:35656686	-0.009	0.027	-0.322	176	0.748
chr6:35656698	-0.045	0.025	-1.814	174	0.071
chr6:35656702	-0.023	0.028	-0.831	174	0.407
chr6:35656704	-0.011	0.025	-0.428	175	0.669
chr6:35656709	-0.009	0.025	-0.37	176	0.712
chr6:35656714	-0.017	0.028	-0.598	175	0.551
chr6:35656722	-0.011	0.023	-0.459	175	0.647
chr6:35656730	-0.016	0.024	-0.648	174	0.518
chr6:35656734	-0.03	0.024	-1.264	176	0.208
chr6:35656737	-0.002	0.026	-0.096	175	0.924
chr6:35656740	-0.001	0.026	-0.045	174	0.964
chr6:35656745	-0.009	0.025	-0.351	175	0.726
chr6:35656754	-0.002	0.021	-0.08	176	0.937
chr6:35656758	-0.01	0.025	-0.417	173	0.677
chr6:35656767	-0.016	0.024	-0.654	178	0.514
chr6:35656784	0.011	0.021	0.526	177	0.6
chr6:35656798	-0.038	0.023	-1.664	166	0.098
chr6:35656811	-0.022	0.023	-0.952	176	0.342
chr6:35656813	-0.013	0.023	-0.565	177	0.573
chr6:35656815	-0.019	0.02	-0.952	177	0.342
chr6:35656823	-0.006	0.022	-0.272	177	0.786
chr6:35656828	-0.016	0.023	-0.729	176	0.467
chr6:35656836	-0.019	0.023	-0.805	176	0.422
chr6:35656848	0.014	0.024	0.562	178	0.575
chr6:35656850	0.015	0.025	0.615	173	0.54
chr6:35656852	0.016	0.024	0.664	174	0.507
chr6:35656855	0.008	0.023	0.343	174	0.732
chr6:35656875	-0.003	0.029	-0.115	177	0.908
chr6:35656906	-0.008	0.033	-0.238	174	0.812
chr6:35656916	-0.041	0.035	-1.163	177	0.246
chr6:35695294	0.013	0.084	0.16	178	0.873
chr6:35695327	0.043	0.074	0.582	171	0.562
chr6:35695346	0.042	0.069	0.609	178	0.543
chr6:35696799	0.01	0.056	0.182	174	0.856
chr6:35704149	0.052	0.041	1.27	171	0.206
chr6:35704224	0.041	0.035	1.188	176	0.236

***Table S2**: Linear mixed model results for FKBP5 methylation on cortisol levels: genomic position, estimate of the regression coefficient, standard error, t-value, degrees of freedom, and p-value.*

CpG (chr6)	Est.	S.E.	*t* val.	d.f.	*p* val.
Chr6:35490599	0.214	0.269	0.795	178	0.428
chr6:35490654	-0.084	0.133	-0.634	177	0.527
chr6:35490693	-0.088	0.137	-0.639	178	0.523
chr6:35490744	0.03	0.146	0.204	178	0.839
chr6:35536880	-0.053	0.299	-0.179	177	0.858
chr6:35536999	0.124	0.263	0.472	178	0.637
chr6:35558386	-0.14	0.205	-0.683	178	0.496
chr6:35558438	-0.436	0.204	-2.137	178	0.034
chr6:35558566	-0.287	0.272	-1.052	178	0.294
chr6:35558710	-0.147	0.269	-0.549	178	0.584
chr6:35558721	-0.093	0.263	-0.354	178	0.724
chr6:35592135	-0.232	0.291	-0.799	176	0.426
chr6:35592141	-0.497	0.267	-1.863	170	0.064
chr6:35592235	-0.071	0.249	-0.287	178	0.774
chr6:35693873	-0.03	0.175	-0.171	178	0.865
chr6:35693881	-0.257	0.149	-1.724	177	0.086
chr6:35693949	-0.307	0.168	-1.83	153	0.069
chr6:35694056	-0.212	0.202	-1.048	123	0.297
chr6:35694626	0.025	0.34	0.074	147	0.941
chr6:35694724	-0.429	0.272	-1.58	178	0.116
chr6:35694748	-0.307	0.267	-1.152	178	0.251
chr6:35694756	-0.165	0.26	-0.632	178	0.528
chr6:35694787	-0.122	0.316	-0.388	178	0.699
chr6:35695192	-0.057	0.196	-0.288	178	0.774
chr6:35695226	0.284	0.289	0.983	177	0.327
chr6:35695240	0.26	0.268	0.968	178	0.335
chr6:35695267	-0.007	0.23	-0.031	36	0.975
chr6:35695270	0.039	0.23	0.17	83	0.866
chr6:35695273	0.165	0.155	1.065	178	0.288
chr6:35696726	-0.215	0.194	-1.109	178	0.269
chr6:35696823	-0.144	0.214	-0.672	178	0.502
chr6:35696870	-0.016	0.215	-0.076	161	0.94
chr6:35697759	-0.209	0.137	-1.521	178	0.13
chr6:35704069	-0.637	0.326	-1.956	178	0.052

Remaining CpGs not considered in the results of this study

CpG (chr6)	Est.	S.E.	*t* val.	d.f.	*p* val.
Chr6:35490674	-0.123	0.091	-1.347	178	0.18
chr6:35490713	-0.076	0.083	-0.913	178	0.362
chr6:35490782	-0.065	0.075	-0.871	178	0.385
chr6:35490787	-0.179	0.087	-2.06	178	0.041
chr6:35490800	0.023	0.098	0.239	178	0.812
chr6:35490812	0.008	0.109	0.078	178	0.938
chr6:35490818	-0.053	0.147	-0.361	175	0.719
chr6:35490820	-0.007	0.154	-0.046	178	0.964
chr6:35490925	-0.121	0.077	-1.567	178	0.119
chr6:35490946	-0.076	0.074	-1.026	178	0.306
chr6:35490965	-0.04	0.071	-0.562	178	0.575
chr6:35558488	-0.454	0.237	-1.92	178	0.056
chr6:35558513	-0.137	0.252	-0.544	178	0.587
chr6:35569751	-0.188	0.119	-1.577	178	0.117
chr6:35569757	-0.18	0.109	-1.65	178	0.101
chr6:35569777	-0.134	0.119	-1.128	178	0.261
chr6:35569896	0.029	0.107	0.266	168	0.79
chr6:35569922	-0.075	0.134	-0.558	178	0.578
chr6:35570224	-0.268	0.221	-1.209	177	0.228
chr6:35578739	-0.228	0.376	-0.608	178	0.544
chr6:35578830	-0.339	0.315	-1.074	178	0.284
chr6:35578891	-0.255	0.348	-0.731	178	0.466
chr6:35592151	-0.18	0.284	-0.633	178	0.527
chr6:35607856	0.113	0.331	0.343	175	0.732
chr6:35607904	-0.001	0.295	-0.005	178	0.996
chr6:35607969	0.214	0.303	0.708	177	0.48
chr6:35608022	0.206	0.33	0.625	176	0.533
chr6:35656649	-0.231	0.079	-2.917	175	0.004
chr6:35656661	-0.076	0.068	-1.118	178	0.265
chr6:35656665	-0.091	0.08	-1.131	177	0.259
chr6:35656667	-0.085	0.078	-1.084	177	0.28
chr6:35656669	-0.064	0.078	-0.822	178	0.412
chr6:35656672	-0.048	0.067	-0.709	178	0.479
chr6:35656679	-0.146	0.075	-1.934	178	0.055
chr6:35656682	-0.088	0.08	-1.107	178	0.27
chr6:35656686	-0.043	0.071	-0.601	178	0.549
chr6:35656698	-0.049	0.067	-0.744	178	0.458
chr6:35656702	-0.084	0.075	-1.122	178	0.264
chr6:35656704	-0.086	0.067	-1.293	178	0.198
chr6:35656709	-0.092	0.067	-1.362	178	0.175
chr6:35656714	-0.08	0.073	-1.101	178	0.272
chr6:35656722	-0.099	0.06	-1.648	177	0.101
chr6:35656730	-0.119	0.063	-1.89	178	0.06
chr6:35656734	-0.073	0.064	-1.151	178	0.251
chr6:35656737	-0.081	0.069	-1.181	178	0.239
chr6:35656740	-0.126	0.068	-1.843	178	0.067
chr6:35656745	-0.043	0.067	-0.641	177	0.523
chr6:35656754	-0.067	0.054	-1.245	178	0.215
chr6:35656758	-0.037	0.066	-0.566	178	0.572
chr6:35656767	-0.056	0.063	-0.88	170	0.38
chr6:35656784	-0.018	0.055	-0.333	175	0.74
chr6:35656798	-0.09	0.06	-1.517	168	0.131
chr6:35656811	-0.031	0.06	-0.513	178	0.609
chr6:35656813	-0.038	0.062	-0.605	178	0.546
chr6:35656815	-0.106	0.052	-2.041	173	0.043
chr6:35656823	-0.078	0.058	-1.341	172	0.182
chr6:35656828	-0.078	0.059	-1.325	175	0.187
chr6:35656836	-0.085	0.059	-1.442	178	0.151
chr6:35656848	-0.076	0.064	-1.186	178	0.237
chr6:35656850	-0.059	0.065	-0.91	178	0.364
chr6:35656852	-0.053	0.064	-0.817	178	0.415
chr6:35656855	-0.041	0.061	-0.67	178	0.504
chr6:35656875	-0.11	0.074	-1.48	160	0.141
chr6:35656906	0.069	0.088	0.786	178	0.433
chr6:35656916	-0.229	0.092	-2.491	178	0.014
chr6:35695294	0.076	0.219	0.347	168	0.729
chr6:35695327	-0.019	0.195	-0.096	178	0.924
chr6:35695346	0.154	0.178	0.865	149	0.388
chr6:35696799	0.087	0.149	0.585	178	0.56
chr6:35704149	0.076	0.109	0.695	178	0.488
chr6:35704224	-0.035	0.092	-0.382	176	0.703

***Table S3**: Linear mixed model results for CTQ score on FKBP5 methylation: genomic position, estimate of the regression coefficient, standard error, t-value, degrees of freedom, and p-value.*

CpG (chr6)	Est.	S.E.	*t* val.	d.f.	*p* val.
chr6:35490599	-0.032	0.017	-1.851	180	0.066
chr6:35490654	0.014	0.036	0.397	183	0.692
chr6:35490693	-0.047	0.035	-1.332	181	0.184
chr6:35490744	-0.019	0.033	-0.586	180	0.559
chr6:35536880	0.001	0.016	0.092	183	0.927
chr6:35536999	-0.021	0.018	-1.136	183	0.257
chr6:35558386	-0.001	0.024	-0.034	183	0.973
chr6:35558438	0.004	0.024	0.168	181	0.867
chr6:35558566	0.005	0.018	0.307	183	0.76
chr6:35558710	0.013	0.018	0.728	180	0.467
chr6:35558721	-0.005	0.018	-0.282	183	0.778
chr6:35592135	-0.005	0.017	-0.321	179	0.749
chr6:35592141	0.014	0.018	0.761	179	0.447
chr6:35592235	0.002	0.02	0.096	183	0.923
chr6:35693873	-0.016	0.028	-0.572	181	0.568
chr6:35693881	-0.063	0.032	-1.986	181	0.049
chr6:35693949	-0.01	0.028	-0.354	180	0.724
chr6:35694056	0.005	0.023	0.208	180	0.836
chr6:35694626	0.023	0.014	1.624	180	0.106
chr6:35694724	-0.003	0.018	-0.178	183	0.859
chr6:35694748	0.024	0.018	1.371	180	0.172
chr6:35694756	0.012	0.018	0.659	180	0.511
chr6:35694787	0.002	0.015	0.107	180	0.915
chr6:35695192	0.031	0.025	1.262	183	0.209
chr6:35695226	0.012	0.017	0.691	181	0.49
chr6:35695240	-0.003	0.018	-0.157	181	0.875
chr6:35695267	0	0.019	-0.002	180	0.998
chr6:35695270	0.004	0.02	0.227	180	0.821
chr6:35695273	0.026	0.031	0.848	181	0.398
chr6:35696726	0.003	0.025	0.133	181	0.894
chr6:35696823	0.004	0.023	0.192	181	0.848
chr6:35696870	-0.017	0.023	-0.77	180	0.443
chr6:35697759	0.032	0.035	0.901	183	0.369
chr6:35704069	0.019	0.014	1.303	180	0.194

Remaining CpGs not considered in the results of this study

CpG (chr6)	Est.	S.E.	*t* val.	d.f.	*p* val.
chr6:35490674	-0.023	0.054	-0.437	183	0.663
chr6:35490713	-0.01	0.056	-0.182	180	0.856
chr6:35490782	0.062	0.064	0.979	180	0.329
chr6:35490787	-0.035	0.057	-0.613	183	0.541
chr6:35490800	-0.011	0.051	-0.222	181	0.825
chr6:35490812	-0.06	0.045	-1.342	183	0.181
chr6:35490818	-0.066	0.033	-2.006	180	0.046
chr6:35490820	-0.046	0.031	-1.455	181	0.147
chr6:35490925	-0.011	0.062	-0.175	180	0.861
chr6:35490946	-0.008	0.064	-0.129	180	0.897
chr6:35490965	0.03	0.067	0.444	180	0.658
chr6:35558488	0.028	0.019	1.476	180	0.142
chr6:35558513	-0.031	0.019	-1.612	180	0.109
chr6:35569751	-0.032	0.04	-0.792	183	0.43
chr6:35569757	-0.035	0.044	-0.796	183	0.427
chr6:35569777	-0.041	0.041	-1.011	183	0.313
chr6:35569896	-0.079	0.044	-1.781	180	0.077
chr6:35569922	-0.027	0.035	-0.765	180	0.446
chr6:35570224	0.008	0.022	0.366	183	0.714
chr6:35578739	-0.006	0.013	-0.469	180	0.64
chr6:35578830	-0.021	0.015	-1.435	180	0.153
chr6:35578891	0.012	0.014	0.896	180	0.372
chr6:35592151	0.001	0.017	0.034	180	0.973
chr6:35607856	0.031	0.014	2.156	180	0.032
chr6:35607904	0.013	0.016	0.809	183	0.42
chr6:35607969	0.003	0.016	0.176	181	0.86
chr6:35608022	0.029	0.015	1.957	183	0.052
chr6:35656649	0.078	0.059	1.317	183	0.189
chr6:35656661	-0.061	0.071	-0.865	183	0.388
chr6:35656665	-0.086	0.06	-1.433	183	0.154
chr6:35656667	-0.061	0.061	-0.987	183	0.325
chr6:35656669	-0.091	0.062	-1.47	183	0.143
chr6:35656672	-0.13	0.072	-1.807	183	0.072
chr6:35656679	-0.016	0.064	-0.25	183	0.803
chr6:35656682	-0.017	0.061	-0.287	183	0.774
chr6:35656686	-0.046	0.068	-0.672	183	0.502
chr6:35656698	-0.025	0.073	-0.348	183	0.728
chr6:35656702	-0.028	0.065	-0.429	183	0.669
chr6:35656704	0.052	0.072	0.715	183	0.475
chr6:35656709	0.049	0.072	0.685	183	0.494
chr6:35656714	0.042	0.066	0.638	183	0.524
chr6:35656722	0.075	0.079	0.944	183	0.346
chr6:35656730	-0.025	0.076	-0.324	181	0.746
chr6:35656734	-0.039	0.076	-0.515	183	0.607
chr6:35656737	-0.102	0.071	-1.437	183	0.152
chr6:35656740	0.071	0.07	1.008	180	0.315
chr6:35656745	-0.054	0.073	-0.733	183	0.465
chr6:35656754	0	0.089	-0.004	183	0.996
chr6:35656758	0.032	0.074	0.432	180	0.666
chr6:35656767	-0.052	0.076	-0.69	180	0.491
chr6:35656784	-0.053	0.087	-0.602	180	0.548
chr6:35656798	0.004	0.08	0.049	180	0.961
chr6:35656811	-0.019	0.081	-0.234	183	0.815
chr6:35656813	-0.004	0.079	-0.051	183	0.959
chr6:35656815	-0.021	0.093	-0.225	180	0.822
chr6:35656823	-0.018	0.084	-0.209	180	0.834
chr6:35656828	0.001	0.082	0.006	180	0.995
chr6:35656836	-0.027	0.079	-0.336	180	0.737
chr6:35656848	0.02	0.076	0.264	180	0.792
chr6:35656850	0.055	0.073	0.752	180	0.453
chr6:35656852	0.01	0.075	0.138	180	0.891
chr6:35656855	-0.015	0.079	-0.189	180	0.85
chr6:35656875	0.07	0.064	1.095	180	0.275
chr6:35656906	-0.029	0.056	-0.529	183	0.597
chr6:35656916	0.079	0.052	1.524	183	0.129
chr6:35695294	0.02	0.022	0.899	180	0.37
chr6:35695327	-0.012	0.025	-0.494	183	0.622
chr6:35695346	-0.024	0.027	-0.902	180	0.368
chr6:35696799	0.011	0.033	0.347	181	0.729
chr6:35704149	-0.015	0.045	-0.326	183	0.745
chr6:35704224	0.063	0.052	1.203	183	0.23

Table S4*: Linear mixed model results for PHQ-9 score on FKBP5 methylation: genomic position, estimate of the regression coefficient, standard error, t-value, degrees of freedom, and p-value.*

CpG (chr6)	Est.	S.E.	*t* val.	d.f.	*p* val.
chr6:35490599	-0.008	0.018	-0.472	177	0.637
chr6:35490654	0.066	0.038	1.749	180	0.082
chr6:35490693	0.057	0.036	1.559	179	0.121
chr6:35490744	0.058	0.034	1.699	178	0.091
chr6:35536880	-0.003	0.017	-0.187	180	0.852
chr6:35536999	0.01	0.019	0.508	180	0.612
chr6:35558386	-0.013	0.025	-0.501	180	0.617
chr6:35558438	0.005	0.024	0.207	179	0.836
chr6:35558566	0.01	0.018	0.568	180	0.571
chr6:35558710	-0.01	0.019	-0.518	180	0.605
chr6:35558721	0.015	0.019	0.795	180	0.427
chr6:35592135	-0.019	0.018	-1.11	177	0.269
chr6:35592141	0.043	0.019	2.303	177	0.022
chr6:35592235	0.002	0.02	0.108	180	0.914
chr6:35693873	-0.032	0.029	-1.118	179	0.265
chr6:35693881	-0.025	0.033	-0.741	178	0.46
chr6:35693949	0.008	0.029	0.289	177	0.773
chr6:35694056	-0.014	0.024	-0.597	177	0.552
chr6:35694626	-0.004	0.013	-0.287	177	0.774
chr6:35694724	-0.015	0.019	-0.82	180	0.413
chr6:35694748	-0.01	0.019	-0.509	178	0.612
chr6:35694756	0.001	0.019	0.067	178	0.947
chr6:35694787	-0.006	0.016	-0.372	178	0.71
chr6:35695192	0.005	0.026	0.175	178	0.861
chr6:35695226	-0.001	0.017	-0.064	178	0.949
chr6:35695240	-0.007	0.018	-0.371	179	0.711
chr6:35695267	-0.001	0.02	-0.04	177	0.968
chr6:35695270	-0.019	0.021	-0.904	177	0.367
chr6:35695273	-0.049	0.032	-1.499	178	0.136
chr6:35696726	-0.008	0.026	-0.307	178	0.759
chr6:35696823	-0.009	0.023	-0.38	178	0.704
chr6:35696870	-0.038	0.023	-1.646	178	0.101
chr6:35697759	0.018	0.037	0.489	180	0.626
chr6:35704069	0.025	0.015	1.693	178	0.092

Remaining CpGs not considered in the results of this study

CpG (chr6)	Est.	S.E.	*t* val.	d.f.	*p* val.
chr6:35490674	0.076	0.055	1.366	180	0.174
chr6:35490713	0.078	0.057	1.369	177	0.173
chr6:35490782	0.084	0.066	1.275	178	0.204
chr6:35490787	0.01	0.059	0.164	180	0.87
chr6:35490800	0.02	0.052	0.383	180	0.702
chr6:35490812	0.068	0.045	1.505	180	0.134
chr6:35490818	0.045	0.034	1.328	178	0.186
chr6:35490820	0.057	0.032	1.771	178	0.078
chr6:35490925	0.015	0.061	0.252	180	0.801
chr6:35490946	0.012	0.066	0.182	177	0.856
chr6:35490965	-0.04	0.069	-0.584	177	0.56
chr6:35558488	-0.004	0.02	-0.212	177	0.832
chr6:35558513	-0.031	0.02	-1.572	178	0.118
chr6:35569751	-0.023	0.042	-0.561	178	0.576
chr6:35569757	-0.002	0.045	-0.04	178	0.968
chr6:35569777	-0.039	0.042	-0.942	178	0.348
chr6:35569896	0.008	0.046	0.17	177	0.865
chr6:35569922	-0.024	0.036	-0.65	177	0.517
chr6:35570224	0.006	0.022	0.285	180	0.776
chr6:35578739	0.009	0.013	0.711	177	0.478
chr6:35578830	-0.021	0.016	-1.354	178	0.178
chr6:35578891	-0.02	0.014	-1.405	177	0.162
chr6:35592151	0.015	0.017	0.887	178	0.376
chr6:35607856	-0.008	0.015	-0.505	178	0.614
chr6:35607904	-0.018	0.017	-1.085	180	0.279
chr6:35607969	0.005	0.017	0.324	178	0.746
chr6:35608022	-0.005	0.015	-0.357	180	0.722
chr6:35656649	0.064	0.061	1.055	180	0.293
chr6:35656661	0.049	0.073	0.672	180	0.502
chr6:35656665	0.054	0.062	0.872	180	0.384
chr6:35656667	0.028	0.064	0.443	180	0.659
chr6:35656669	0.001	0.065	0.011	180	0.992
chr6:35656672	-0.013	0.075	-0.177	180	0.86
chr6:35656679	0.025	0.067	0.38	180	0.704
chr6:35656682	0.049	0.063	0.77	180	0.442
chr6:35656686	0.041	0.071	0.584	180	0.56
chr6:35656698	-0.003	0.076	-0.038	180	0.969
chr6:35656702	-0.012	0.068	-0.181	180	0.857
chr6:35656704	0.085	0.075	1.13	180	0.26
chr6:35656709	0.012	0.075	0.156	180	0.876
chr6:35656714	-0.006	0.069	-0.088	180	0.93
chr6:35656722	-0.012	0.083	-0.15	180	0.881
chr6:35656730	-0.001	0.08	-0.008	178	0.994
chr6:35656734	0.01	0.079	0.129	180	0.897
chr6:35656737	-0.021	0.074	-0.286	180	0.775
chr6:35656740	-0.015	0.074	-0.201	178	0.841
chr6:35656745	0.011	0.076	0.142	180	0.888
chr6:35656754	0.001	0.093	0.009	180	0.993
chr6:35656758	0.092	0.076	1.199	178	0.232
chr6:35656767	-0.053	0.078	-0.679	178	0.498
chr6:35656784	-0.069	0.091	-0.756	178	0.45
chr6:35656798	-0.009	0.083	-0.104	177	0.917
chr6:35656811	0.078	0.084	0.936	180	0.351
chr6:35656813	-0.061	0.081	-0.753	180	0.452
chr6:35656815	-0.003	0.095	-0.035	178	0.972
chr6:35656823	-0.003	0.087	-0.037	178	0.97
chr6:35656828	0.029	0.084	0.345	178	0.73
chr6:35656836	-0.013	0.082	-0.164	177	0.87
chr6:35656848	0.052	0.078	0.663	178	0.508
chr6:35656850	0.038	0.076	0.493	177	0.623
chr6:35656852	-0.013	0.077	-0.164	178	0.87
chr6:35656855	0.033	0.081	0.402	177	0.688
chr6:35656875	0.059	0.066	0.889	178	0.375
chr6:35656906	-0.053	0.058	-0.926	180	0.356
chr6:35656916	0.04	0.054	0.729	180	0.467
chr6:35695294	-0.009	0.023	-0.394	178	0.694
chr6:35695327	-0.02	0.025	-0.822	180	0.412
chr6:35695346	-0.005	0.026	-0.195	177	0.846
chr6:35696799	-0.024	0.034	-0.707	178	0.481
chr6:35704149	-0.006	0.047	-0.118	180	0.906
chr6:35704224	-0.007	0.055	-0.119	180	0.905

Interaction models

***Table S5**: Linear mixed model results for FKBP5 methylation x SNP interaction on FKBP5 expression: genomic position, estimate of the regression coefficient, standard error, t-value, degrees of freedom, and p-value.*

CpG (chr6)	Est.	S.E.	*t* val.	d.f.	*p* val.
chr6:35490599	0.178	0.22	0.806	179	0.421
chr6:35490654	0.031	0.115	0.27	179	0.787
chr6:35490693	0.054	0.123	0.443	179	0.659
chr6:35490744	0.136	0.126	1.077	179	0.283
chr6:35536880	-0.151	0.247	-0.612	179	0.541
chr6:35536999	-0.041	0.217	-0.187	178	0.852
chr6:35558386	-0.2	0.11	-1.824	179	0.07
chr6:35558438	-0.075	0.102	-0.732	180	0.465
chr6:35558566	-0.187	0.189	-0.989	180	0.324
chr6:35558710	-0.115	0.182	-0.632	179	0.528
chr6:35558721	-0.085	0.182	-0.464	179	0.643
chr6:35592135	-0.171	0.254	-0.674	177	0.501
chr6:35592141	0.104	0.22	0.472	178	0.637
chr6:35592235	-0.007	0.149	-0.046	179	0.963
chr6:35693873	-0.204	0.149	-1.371	179	0.172
chr6:35693881	0.128	0.124	1.028	178	0.305
chr6:35693949	-0.054	0.103	-0.529	178	0.597
chr6:35694056	-0.082	0.15	-0.544	178	0.587
chr6:35694626	-0.117	0.263	-0.447	178	0.656
chr6:35694724	-0.051	0.196	-0.26	178	0.795
chr6:35694748	0.125	0.215	0.58	178	0.563
chr6:35694756	-0.009	0.198	-0.048	178	0.962
chr6:35694787	-0.069	0.246	-0.279	179	0.781
chr6:35695192	0.033	0.153	0.218	178	0.828
chr6:35695226	-0.148	0.186	-0.796	179	0.427
chr6:35695240	-0.134	0.171	-0.785	179	0.434
chr6:35695267	0.076	0.184	0.412	179	0.681
chr6:35695270	0.069	0.184	0.373	179	0.71
chr6:35695273	0.058	0.128	0.451	179	0.653
chr6:35696726	0.104	0.166	0.625	179	0.533
chr6:35696823	-0.205	0.173	-1.182	179	0.239
chr6:35696870	0.033	0.165	0.201	179	0.841
chr6:35697759	-0.062	0.122	-0.507	179	0.613
chr6:35704069	-0.142	0.213	-0.668	178	0.505

Remaining CpGs not considered in the results of this study

CpG (chr6)	Est.	S.E.	*t* val.	d.f.	*p* val.
chr6:35490674	0.086	0.078	1.099	179	0.273
chr6:35490713	-0.009	0.072	-0.126	178	0.9
chr6:35490782	-0.002	0.065	-0.036	179	0.972
chr6:35490787	0.042	0.074	0.57	179	0.569
chr6:35490800	0.102	0.079	1.295	178	0.197
chr6:35490812	0.047	0.087	0.543	178	0.588
chr6:35490818	0.049	0.117	0.422	179	0.674
chr6:35490820	0.135	0.128	1.061	179	0.29
chr6:35490925	-0.01	0.067	-0.149	178	0.882
chr6:35490946	0.002	0.065	0.038	179	0.97
chr6:35490965	0.015	0.064	0.232	179	0.817
chr6:35558488	0.035	0.207	0.168	178	0.867
chr6:35558513	-0.113	0.213	-0.532	179	0.595
chr6:35569751	-0.104	0.081	-1.286	179	0.2
chr6:35569757	-0.083	0.074	-1.114	179	0.267
chr6:35569777	-0.1	0.079	-1.262	179	0.209
chr6:35569896	-0.143	0.083	-1.717	180	0.088
chr6:35569922	-0.161	0.103	-1.551	180	0.123
chr6:35570224	-0.098	0.116	-0.852	179	0.395
chr6:35578739	-0.333	0.318	-1.045	178	0.298
chr6:35578830	-0.066	0.286	-0.232	180	0.817
chr6:35578891	0.089	0.225	0.396	180	0.692
chr6:35592151	0.12	0.238	0.507	179	0.613
chr6:35607856	-0.063	0.282	-0.224	178	0.823
chr6:35607904	-0.119	0.247	-0.484	178	0.629
chr6:35607969	0.327	0.238	1.372	179	0.172
chr6:35608022	-0.116	0.255	-0.455	179	0.65
chr6:35656649	0.019	0.07	0.272	179	0.786
chr6:35656661	-0.003	0.058	-0.044	179	0.965
chr6:35656665	0.016	0.07	0.232	179	0.817
chr6:35656667	0.053	0.067	0.799	179	0.425
chr6:35656669	0.011	0.068	0.164	179	0.87
chr6:35656672	0.029	0.059	0.493	178	0.623
chr6:35656679	-0.046	0.066	-0.697	179	0.487
chr6:35656682	0.039	0.071	0.548	179	0.585
chr6:35656686	0.012	0.062	0.2	178	0.842
chr6:35656698	0.013	0.058	0.231	178	0.818
chr6:35656702	-0.032	0.065	-0.493	179	0.623
chr6:35656704	-0.021	0.058	-0.356	180	0.722
chr6:35656709	0.035	0.059	0.584	179	0.56
chr6:35656714	0.029	0.063	0.462	179	0.644
chr6:35656722	0.014	0.054	0.258	179	0.797
chr6:35656730	0.087	0.056	1.559	179	0.121
chr6:35656734	0.022	0.056	0.388	179	0.698
chr6:35656737	0.077	0.06	1.283	180	0.201
chr6:35656740	0.026	0.061	0.419	181	0.676
chr6:35656745	0.039	0.057	0.684	179	0.495
chr6:35656754	0.04	0.048	0.832	179	0.406
chr6:35656758	-0.01	0.058	-0.18	179	0.858
chr6:35656767	0.027	0.055	0.487	180	0.627
chr6:35656784	0.025	0.048	0.525	180	0.6
chr6:35656798	0.015	0.051	0.294	179	0.769
chr6:35656811	0.022	0.052	0.423	179	0.673
chr6:35656813	0.056	0.054	1.041	178	0.299
chr6:35656815	0.059	0.045	1.31	178	0.192
chr6:35656823	0.005	0.049	0.103	178	0.918
chr6:35656828	0.03	0.053	0.563	178	0.574
chr6:35656836	0.077	0.052	1.491	178	0.138
chr6:35656848	0.004	0.054	0.078	179	0.938
chr6:35656850	-0.026	0.057	-0.448	178	0.655
chr6:35656852	-0.016	0.057	-0.282	179	0.778
chr6:35656855	-0.021	0.053	-0.402	179	0.688
chr6:35656875	0.025	0.067	0.375	178	0.708
chr6:35656906	0.044	0.075	0.589	179	0.556
chr6:35656916	0.036	0.085	0.423	179	0.673
chr6:35695294	0.03	0.169	0.177	179	0.86
chr6:35695327	-0.019	0.159	-0.119	180	0.906
chr6:35695346	0.13	0.143	0.912	179	0.363
chr6:35696799	0.002	0.127	0.013	179	0.99
chr6:35704149	-0.017	0.092	-0.181	178	0.856
chr6:35704224	0.117	0.079	1.487	180	0.139

Table S6*: Linear mixed model results for FKBP5 methylation x SNP interaction on cortisol: genomic position, estimate of the regression coefficient, standard error, t-value, degrees of freedom, and p-value.*

CpG (chr6)	Est.	S.E.	*t* val.	d.f.	*p* val.
chr6:35490599	0.327	0.54	0.606	180	0.545
chr6:35490654	0.095	0.285	0.334	180	0.739
chr6:35490693	0.125	0.301	0.415	180	0.678
chr6:35490744	0.309	0.311	0.995	180	0.321
chr6:35536880	0.044	0.609	0.073	180	0.942
chr6:35536999	0.129	0.537	0.24	180	0.81
chr6:35558386	-0.344	0.278	-1.239	180	0.217
chr6:35558438	-0.088	0.25	-0.35	180	0.727
chr6:35558566	-0.666	0.46	-1.449	180	0.149
chr6:35558710	-0.54	0.447	-1.209	180	0.228
chr6:35558721	-0.28	0.449	-0.625	180	0.533
chr6:35592135	-0.476	0.614	-0.775	178	0.439
chr6:35592141	0.377	0.548	0.688	179	0.493
chr6:35592235	-0.18	0.365	-0.493	180	0.623
chr6:35693873	-0.408	0.369	-1.108	180	0.27
chr6:35693881	-0.517	0.303	-1.706	180	0.09
chr6:35693949	-0.121	0.255	-0.472	180	0.637
chr6:35694056	-0.15	0.378	-0.396	180	0.693
chr6:35694626	0.117	0.65	0.18	180	0.857
chr6:35694724	-0.193	0.489	-0.395	180	0.693
chr6:35694748	0.105	0.534	0.196	180	0.845
chr6:35694756	0.015	0.494	0.031	180	0.975
chr6:35694787	-0.935	0.608	-1.538	180	0.126
chr6:35695192	0.515	0.375	1.371	180	0.172
chr6:35695226	-0.275	0.456	-0.604	180	0.547
chr6:35695240	-0.022	0.422	-0.052	180	0.959
chr6:35695267	0.556	0.457	1.217	180	0.225
chr6:35695270	0.328	0.457	0.719	180	0.473
chr6:35695273	0.184	0.317	0.582	180	0.561
chr6:35696726	-0.608	0.406	-1.498	180	0.136
chr6:35696823	-0.685	0.425	-1.611	180	0.109
chr6:35696870	-0.595	0.403	-1.476	180	0.142
chr6:35697759	-0.533	0.296	-1.797	180	0.074
chr6:35704069	0.581	0.518	1.121	180	0.264

Remaining CpGs not considered in the results of this study

CpG (chr6)	Est.	S.E.	*t* val.	d.f.	*p* val.
chr6:35490674	-0.069	0.193	-0.357	180	0.721
chr6:35490713	-0.205	0.179	-1.145	180	0.254
chr6:35490782	0.055	0.16	0.341	180	0.734
chr6:35490787	0.202	0.179	1.123	180	0.263
chr6:35490800	0.071	0.196	0.362	180	0.718
chr6:35490812	-0.116	0.216	-0.539	180	0.591
chr6:35490818	-0.157	0.289	-0.545	180	0.587
chr6:35490820	0.239	0.315	0.761	180	0.448
chr6:35490925	-0.003	0.165	-0.019	180	0.985
chr6:35490946	0.038	0.159	0.238	180	0.812
chr6:35490965	0.098	0.157	0.623	180	0.534
chr6:35558488	0.163	0.509	0.319	180	0.75
chr6:35558513	-0.9	0.529	-1.703	180	0.09
chr6:35569751	-0.283	0.197	-1.434	180	0.153
chr6:35569757	-0.279	0.181	-1.546	180	0.124
chr6:35569777	-0.35	0.193	-1.817	180	0.071
chr6:35569896	-0.233	0.203	-1.143	180	0.255
chr6:35569922	-0.393	0.252	-1.559	180	0.121
chr6:35570224	-0.481	0.288	-1.669	180	0.097
chr6:35578739	-0.472	0.792	-0.597	180	0.552
chr6:35578830	-0.477	0.694	-0.687	180	0.493
chr6:35578891	-0.11	0.556	-0.197	180	0.844
chr6:35592151	-0.25	0.583	-0.428	180	0.669
chr6:35607856	-0.27	0.695	-0.389	180	0.698
chr6:35607904	0.4	0.61	0.655	180	0.513
chr6:35607969	0.021	0.592	0.036	180	0.971
chr6:35608022	-0.199	0.629	-0.317	180	0.752
chr6:35656649	0.129	0.171	0.752	180	0.453
chr6:35656661	0.063	0.143	0.439	180	0.661
chr6:35656665	-0.006	0.173	-0.035	180	0.972
chr6:35656667	0.263	0.162	1.618	180	0.107
chr6:35656669	0.243	0.167	1.458	180	0.146
chr6:35656672	0.166	0.145	1.141	180	0.255
chr6:35656679	0.152	0.162	0.934	180	0.352
chr6:35656682	0.119	0.173	0.689	180	0.492
chr6:35656686	0.07	0.153	0.46	180	0.646
chr6:35656698	0.157	0.144	1.086	180	0.279
chr6:35656702	0.087	0.16	0.541	180	0.589
chr6:35656704	0.149	0.141	1.056	180	0.292
chr6:35656709	0.09	0.145	0.623	180	0.534
chr6:35656714	0.206	0.154	1.339	180	0.182
chr6:35656722	0.082	0.132	0.624	180	0.533
chr6:35656730	0.335	0.134	2.491	180	0.014
chr6:35656734	0.225	0.137	1.642	180	0.102
chr6:35656737	0.044	0.148	0.296	180	0.768
chr6:35656740	0.062	0.146	0.422	180	0.674
chr6:35656745	0.078	0.14	0.556	180	0.579
chr6:35656754	0.127	0.118	1.08	180	0.282
chr6:35656758	0.159	0.143	1.115	180	0.266
chr6:35656767	0.09	0.135	0.669	180	0.505
chr6:35656784	0.133	0.117	1.137	180	0.257
chr6:35656798	0.083	0.126	0.662	180	0.509
chr6:35656811	0.101	0.128	0.793	180	0.429
chr6:35656813	0.056	0.133	0.418	180	0.676
chr6:35656815	0.037	0.112	0.331	180	0.741
chr6:35656823	0	0.12	0	180	1
chr6:35656828	0.134	0.131	1.022	180	0.308
chr6:35656836	0.274	0.126	2.176	180	0.031
chr6:35656848	0.055	0.134	0.41	180	0.682
chr6:35656850	0.172	0.14	1.232	180	0.219
chr6:35656852	0.044	0.14	0.314	180	0.754
chr6:35656855	0.076	0.129	0.592	180	0.555
chr6:35656875	0.111	0.163	0.678	180	0.499
chr6:35656906	0.271	0.181	1.492	180	0.137
chr6:35656916	0.266	0.208	1.276	180	0.204
chr6:35695294	0.202	0.418	0.483	180	0.63
chr6:35695327	0.145	0.389	0.373	180	0.71
chr6:35695346	0.42	0.348	1.209	180	0.228
chr6:35696799	-0.291	0.316	-0.92	180	0.359
chr6:35704149	0.12	0.229	0.525	180	0.6
chr6:35704224	0.019	0.193	0.1	180	0.921

***Table S7**: Linear mixed model results for CTQ x SNP interaction on FKBP5 methylation: genomic position, estimate of the regression coefficient, standard error, t-value, degrees of freedom, and p-value.*

CpG (chr6)	Est.	S.E.	*t* val.	d.f.	*p* val.
Chr6:35490599	-0.07	0.033	-2.133	179	0.034
chr6:35490654	-0.053	0.071	-0.751	181	0.454
chr6:35490693	-0.067	0.068	-0.983	180	0.327
chr6:35490744	-0.046	0.064	-0.722	181	0.471
chr6:35536880	-0.037	0.032	-1.166	181	0.245
chr6:35536999	-0.026	0.036	-0.717	181	0.474
chr6:35558386	0.015	0.047	0.312	181	0.756
chr6:35558438	0.025	0.046	0.538	180	0.591
chr6:35558566	0.008	0.035	0.24	181	0.81
chr6:35558710	0.043	0.035	1.234	180	0.219
chr6:35558721	0.03	0.035	0.844	181	0.4
chr6:35592135	-0.033	0.033	-0.996	179	0.321
chr6:35592141	-0.028	0.036	-0.787	178	0.432
chr6:35592235	0.033	0.038	0.855	181	0.393
chr6:35693873	-0.039	0.053	-0.728	180	0.467
chr6:35693881	-0.016	0.061	-0.264	180	0.792
chr6:35693949	-0.027	0.056	-0.491	178	0.624
chr6:35694056	-0.036	0.046	-0.793	179	0.429
chr6:35694626	0.027	0.027	1	179	0.319
chr6:35694724	0.082	0.034	2.387	181	0.018
chr6:35694748	0.097	0.034	2.829	179	0.005
chr6:35694756	0.103	0.035	2.955	179	0.004
chr6:35694787	0.057	0.029	1.938	179	0.054
chr6:35695192	0.026	0.048	0.539	181	0.591
chr6:35695226	0.025	0.032	0.8	180	0.425
chr6:35695240	0.049	0.033	1.464	180	0.145
chr6:35695267	0.011	0.036	0.311	178	0.756
chr6:35695270	0.023	0.038	0.599	178	0.55
chr6:35695273	0.049	0.061	0.806	180	0.421
chr6:35696726	0.076	0.048	1.581	180	0.116
chr6:35696823	0.07	0.044	1.607	180	0.11
chr6:35696870	0.067	0.044	1.534	179	0.127
chr6:35697759	0.138	0.064	2.151	181	0.033
chr6:35704069	0.01	0.028	0.353	179	0.724

Remaining CpGs not considered in the results of this study

CpG (chr6)	Est.	S.E.	*t* val.	d.f.	*p* val.
Chr6:35490674	-0.112	0.104	-1.08	181	0.281
chr6:35490713	-0.089	0.109	-0.816	179	0.416
chr6:35490782	-0.062	0.124	-0.499	179	0.618
chr6:35490787	-0.225	0.109	-2.058	181	0.041
chr6:35490800	0.087	0.099	0.879	181	0.38
chr6:35490812	-0.039	0.087	-0.452	181	0.652
chr6:35490818	-0.036	0.065	-0.558	179	0.577
chr6:35490820	0.003	0.061	0.045	181	0.964
chr6:35490925	-0.079	0.12	-0.661	180	0.51
chr6:35490946	0.13	0.124	1.048	179	0.296
chr6:35490965	0.216	0.129	1.673	179	0.096
chr6:35558488	-0.063	0.037	-1.718	178	0.087
chr6:35558513	0.001	0.037	0.023	179	0.982
chr6:35569751	0.163	0.078	2.078	181	0.039
chr6:35569757	0.105	0.086	1.226	181	0.222
chr6:35569777	0.159	0.079	2.016	181	0.045
chr6:35569896	0.081	0.086	0.944	179	0.347
chr6:35569922	0.127	0.067	1.883	178	0.061
chr6:35570224	0.1	0.042	2.368	181	0.019
chr6:35578739	-0.015	0.025	-0.617	179	0.538
chr6:35578830	0.007	0.029	0.246	179	0.806
chr6:35578891	-0.024	0.026	-0.922	179	0.358
chr6:35592151	0.003	0.033	0.079	179	0.937
chr6:35607856	-0.021	0.028	-0.746	180	0.457
chr6:35607904	-0.044	0.032	-1.376	181	0.17
chr6:35607969	-0.006	0.032	-0.197	180	0.844
chr6:35608022	0.01	0.028	0.36	181	0.719
chr6:35656649	0.01	0.116	0.089	181	0.929
chr6:35656661	-0.016	0.138	-0.115	181	0.909
chr6:35656665	0.096	0.116	0.828	181	0.409
chr6:35656667	0.113	0.119	0.95	181	0.343
chr6:35656669	0.006	0.121	0.052	181	0.959
chr6:35656672	0.011	0.141	0.076	181	0.94
chr6:35656679	0.004	0.126	0.03	181	0.976
chr6:35656682	-0.002	0.119	-0.014	181	0.989
chr6:35656686	0.052	0.133	0.395	181	0.694
chr6:35656698	-0.174	0.142	-1.228	181	0.221
chr6:35656702	-0.035	0.128	-0.277	181	0.782
chr6:35656704	-0.215	0.14	-1.537	181	0.126
chr6:35656709	-0.159	0.14	-1.142	181	0.255
chr6:35656714	-0.041	0.129	-0.317	181	0.751
chr6:35656722	-0.142	0.155	-0.915	181	0.362
chr6:35656730	-0.216	0.148	-1.457	181	0.147
chr6:35656734	-0.18	0.147	-1.22	181	0.224
chr6:35656737	-0.232	0.138	-1.681	181	0.094
chr6:35656740	-0.169	0.137	-1.235	180	0.218
chr6:35656745	-0.169	0.142	-1.185	181	0.238
chr6:35656754	-0.28	0.172	-1.625	181	0.106
chr6:35656758	-0.074	0.145	-0.508	180	0.612
chr6:35656767	-0.044	0.148	-0.296	179	0.768
chr6:35656784	-0.311	0.17	-1.831	180	0.069
chr6:35656798	-0.257	0.156	-1.651	179	0.1
chr6:35656811	-0.099	0.157	-0.632	181	0.528
chr6:35656813	-0.163	0.153	-1.066	181	0.288
chr6:35656815	-0.313	0.179	-1.748	180	0.082
chr6:35656823	-0.257	0.163	-1.578	179	0.116
chr6:35656828	-0.285	0.158	-1.805	180	0.073
chr6:35656836	-0.135	0.155	-0.872	179	0.384
chr6:35656848	-0.195	0.147	-1.326	181	0.186
chr6:35656850	-0.178	0.144	-1.24	179	0.217
chr6:35656852	-0.334	0.144	-2.322	179	0.021
chr6:35656855	-0.189	0.154	-1.229	179	0.221
chr6:35656875	-0.197	0.124	-1.586	179	0.115
chr6:35656906	-0.049	0.106	-0.459	180	0.647
chr6:35656916	-0.024	0.099	-0.239	181	0.811
chr6:35695294	0.044	0.042	1.046	179	0.297
chr6:35695327	-0.006	0.048	-0.133	181	0.894
chr6:35695346	0.031	0.052	0.598	179	0.551
chr6:35696799	0.02	0.064	0.313	180	0.755
chr6:35704149	0	0.087	0.001	181	0.999
chr6:35704224	-0.222	0.101	-2.207	181	0.029

***Table S8**: Linear mixed model results for PHQ-9 x SNP interaction on FKBP5 methylation: genomic position, estimate of the regression coefficient, standard error, t-value, degrees of freedom, and p-value.*

CpG (chr6)	Est.	S.E.	*t* val.	d.f.	*p* val.
chr6:35490599	-0.031	0.034	-0.903	176	0.368
chr6:35490654	0.008	0.071	0.119	178	0.905
chr6:35490693	-0.073	0.068	-1.081	178	0.281
chr6:35490744	0.001	0.064	0.019	178	0.985
chr6:35536880	0.011	0.032	0.353	178	0.725
chr6:35536999	0.041	0.036	1.141	178	0.255
chr6:35558386	0.122	0.046	2.62	178	0.01
chr6:35558438	0.114	0.045	2.502	177	0.013
chr6:35558566	0.023	0.034	0.663	178	0.508
chr6:35558710	0.017	0.035	0.49	178	0.625
chr6:35558721	0.037	0.035	1.047	178	0.297
chr6:35592135	0.037	0.033	1.123	176	0.263
chr6:35592141	0.027	0.035	0.762	175	0.447
chr6:35592235	0.009	0.038	0.239	178	0.811
chr6:35693873	-0.006	0.054	-0.119	178	0.906
chr6:35693881	0.023	0.062	0.374	177	0.709
chr6:35693949	-0.075	0.056	-1.346	176	0.18
chr6:35694056	-0.093	0.045	-2.047	176	0.042
chr6:35694626	0.002	0.026	0.086	176	0.931
chr6:35694724	0.033	0.035	0.933	178	0.352
chr6:35694748	0.015	0.036	0.416	177	0.678
chr6:35694756	0.016	0.036	0.46	177	0.646
chr6:35694787	0.004	0.03	0.123	177	0.902
chr6:35695192	-0.079	0.048	-1.643	177	0.102
chr6:35695226	0.004	0.032	0.12	177	0.905
chr6:35695240	-0.006	0.034	-0.174	177	0.862
chr6:35695267	-0.022	0.037	-0.608	175	0.544
chr6:35695270	-0.008	0.038	-0.197	176	0.844
chr6:35695273	0.04	0.061	0.657	178	0.512
chr6:35696726	0.059	0.049	1.213	178	0.227
chr6:35696823	0.054	0.044	1.22	177	0.224
chr6:35696870	0.059	0.044	1.353	177	0.178
chr6:35697759	0.005	0.066	0.071	178	0.943
chr6:35704069	0.012	0.028	0.439	177	0.661

Remaining CpGs not considered in the results of this study

CpG (chr6)	Est.	S.E.	*t* val.	d.f.	*p* val.
chr6:35490674	-0.07	0.104	-0.675	178	0.501
chr6:35490713	-0.078	0.109	-0.722	176	0.471
chr6:35490782	-0.155	0.124	-1.243	177	0.215
chr6:35490787	-0.139	0.111	-1.254	178	0.211
chr6:35490800	0.076	0.097	0.776	178	0.439
chr6:35490812	-0.099	0.085	-1.165	178	0.245
chr6:35490818	-0.033	0.065	-0.516	177	0.607
chr6:35490820	-0.043	0.061	-0.704	178	0.482
chr6:35490925	-0.079	0.114	-0.699	178	0.486
chr6:35490946	-0.014	0.123	-0.118	177	0.906
chr6:35490965	-0.034	0.128	-0.267	177	0.789
chr6:35558488	0.01	0.038	0.274	176	0.784
chr6:35558513	0.01	0.037	0.27	176	0.787
chr6:35569751	0.079	0.079	1.011	177	0.313
chr6:35569757	0.095	0.086	1.101	177	0.272
chr6:35569777	0.082	0.079	1.036	178	0.302
chr6:35569896	0.12	0.087	1.377	176	0.17
chr6:35569922	0.062	0.069	0.907	176	0.366
chr6:35570224	0.05	0.041	1.211	178	0.227
chr6:35578739	0.03	0.025	1.193	176	0.235
chr6:35578830	-0.041	0.029	-1.436	176	0.153
chr6:35578891	-0.007	0.026	-0.27	176	0.787
chr6:35592151	0.031	0.032	0.949	176	0.344
chr6:35607856	-0.022	0.029	-0.75	177	0.454
chr6:35607904	-0.043	0.032	-1.341	178	0.182
chr6:35607969	-0.02	0.032	-0.613	177	0.541
chr6:35608022	0.007	0.029	0.26	178	0.795
chr6:35656649	0.097	0.116	0.843	178	0.4
chr6:35656661	-0.178	0.137	-1.293	178	0.198
chr6:35656665	-0.192	0.115	-1.668	178	0.097
chr6:35656667	-0.188	0.118	-1.585	178	0.115
chr6:35656669	-0.289	0.121	-2.392	178	0.018
chr6:35656672	-0.232	0.141	-1.646	178	0.101
chr6:35656679	-0.193	0.126	-1.533	178	0.127
chr6:35656682	-0.219	0.119	-1.839	178	0.068
chr6:35656686	-0.238	0.133	-1.795	178	0.074
chr6:35656698	-0.3	0.143	-2.106	178	0.037
chr6:35656702	-0.173	0.128	-1.348	178	0.179
chr6:35656704	-0.188	0.141	-1.332	178	0.185
chr6:35656709	-0.289	0.14	-2.061	178	0.041
chr6:35656714	-0.147	0.13	-1.127	178	0.261
chr6:35656722	-0.306	0.155	-1.973	178	0.05
chr6:35656730	-0.294	0.15	-1.967	178	0.051
chr6:35656734	-0.167	0.149	-1.12	178	0.264
chr6:35656737	-0.279	0.139	-2.002	178	0.047
chr6:35656740	-0.242	0.138	-1.751	178	0.082
chr6:35656745	-0.153	0.144	-1.06	178	0.291
chr6:35656754	-0.301	0.174	-1.729	178	0.086
chr6:35656758	-0.213	0.144	-1.476	178	0.142
chr6:35656767	-0.126	0.147	-0.86	177	0.391
chr6:35656784	-0.326	0.171	-1.91	177	0.058
chr6:35656798	-0.376	0.155	-2.42	177	0.017
chr6:35656811	-0.206	0.157	-1.313	178	0.191
chr6:35656813	-0.27	0.152	-1.774	178	0.078
chr6:35656815	-0.473	0.177	-2.68	177	0.008
chr6:35656823	-0.273	0.164	-1.666	177	0.098
chr6:35656828	-0.338	0.157	-2.154	177	0.033
chr6:35656836	-0.302	0.154	-1.956	176	0.052
chr6:35656848	-0.205	0.146	-1.402	178	0.163
chr6:35656850	-0.223	0.144	-1.544	177	0.124
chr6:35656852	-0.318	0.144	-2.201	178	0.029
chr6:35656855	-0.215	0.154	-1.392	176	0.166
chr6:35656875	-0.264	0.124	-2.137	176	0.034
chr6:35656906	-0.019	0.107	-0.176	178	0.86
chr6:35656916	-0.093	0.1	-0.927	178	0.355
chr6:35695294	-0.024	0.043	-0.551	177	0.582
chr6:35695327	-0.056	0.047	-1.191	178	0.235
chr6:35695346	-0.064	0.049	-1.307	176	0.193
chr6:35696799	0.016	0.065	0.252	177	0.801
chr6:35704149	-0.061	0.088	-0.687	178	0.493
chr6:35704224	0.102	0.103	0.985	178	0.326
